# Occurrence of mental illness following prenatal and early childhood exposure to tetrachloroethylene (PCE)-contaminated drinking water: a retrospective cohort study

**DOI:** 10.1186/1476-069X-11-2

**Published:** 2012-01-20

**Authors:** Ann Aschengrau, Janice M Weinberg, Patricia A Janulewicz, Megan E Romano, Lisa G Gallagher, Michael R Winter, Brett R Martin, Veronica M Vieira, Thomas F Webster, Roberta F White, David M Ozonoff

**Affiliations:** 1 Department of Epidemiology, Boston University School of Public Health, Talbot 3E, 715 Albany Street, Boston, MA 02118, USA; 2 Department of Biostatistics, Boston University School of Public Health, Crosstown, 715 Albany Street, Boston, MA 02118, USA; 3 Department of Epidemiology, University of Washington, Box 357236, Seattle WA, 98195, USA; 4 Department of Environmental Health, Boston University School of Public Health, Talbot 4W, 715 Albany Street, Boston, MA 02118, USA; 5 Data Coordinating Center, Boston University School of Public Health, Crosstown, 715 Albany Street, Boston MA 02118, USA; 6 Department of Neurology, Boston University School of Medicine, 72 East Concord Street, Boston MA 02118, USA

## Abstract

**Background:**

While many studies of adults with solvent exposure have shown increased risks of anxiety and depressive disorders, there is little information on the impact of prenatal and early childhood exposure on the subsequent risk of mental illness. This retrospective cohort study examined whether early life exposure to tetrachloroethylene (PCE)-contaminated drinking water influenced the occurrence of depression, bipolar disorder, post-traumatic stress disorder, and schizophrenia among adults from Cape Cod, Massachusetts.

**Methods:**

A total of 1,512 subjects born between 1969 and 1983 were studied, including 831 subjects with both prenatal and early childhood PCE exposure and 547 unexposed subjects. Participants completed questionnaires to gather information on mental illnesses, demographic and medical characteristics, other sources of solvent exposure, and residences from birth through 1990. PCE exposure originating from the vinyl-liner of water distribution pipes was assessed using water distribution system modeling software that incorporated a leaching and transport algorithm.

**Results:**

No meaningful increases in risk ratios (RR) for depression were observed among subjects with prenatal and early childhood exposure (RR: 1.1, 95% CI: 0.9-1.4). However, subjects with prenatal and early childhood exposure had a 1.8-fold increased risk of bipolar disorder (N = 36 exposed cases, 95% CI: 0.9-1.4), a 1.5-fold increased risk post-traumatic stress disorder (N = 47 exposed cases, 95% CI: 0.9-2.5), and a 2.1-fold increased risk of schizophrenia (N = 3 exposed cases, 95% CI: 0.2-20.0). Further increases in the risk ratio were observed for bipolar disorder (N = 18 exposed cases, RR; 2.7, 95% CI: 1.3-5.6) and post-traumatic stress disorder (N = 18 exposed cases, RR: 1.7, 95% CI: 0.9-3.2) among subjects with the highest exposure levels.

**Conclusions:**

The results of this study provide evidence against an impact of early life exposure to PCE on the risk of depression. In contrast, the results provide support for an impact of early life exposure on the risk of bipolar disorder and post-traumatic stress disorder. The number of schizophrenia cases was too small to draw reliable conclusions. These findings should be confirmed in investigations of other similarly exposed populations.

## Background

From the late 1960s through early 1980 public water companies in Massachusetts installed vinyl-lined (VL) asbestos-cement (AC) water pipes to solve alkalinity problems in dead end sections of their distribution systems [1]. The liner was applied by spraying a mixture of vinyl resin (Piccotex™) and tetrachloroethylene (PCE) onto the inner pipe surface. Because the pipes were dried for 48 hours before shipping [Demond AH: A source of tetrachloroethylene in the drinking water of New England: an evaluation of toxicity of tetrachloroethylene and the prediction of its leaching rates from vinyl-lined asbestos-cement pipe. MS Thesis. Massachusetts Institute of Technology; 1982], it was assumed that most of the PCE would evaporate before installation. However, more than a decade lapsed before government officials learned that large quantities of PCE had remained in the liner and were leaching into the public drinking water supplies.

Surveys conducted in 1980 determined that approximately 660 miles of VL/AC pipes had been installed across Massachusetts; a large proportion was placed in the Cape Cod region [2]. Drinking water supplies on Cape Cod had PCE levels ranging from 1.5 to 7,750 μg/L. Because replacing the pipes was prohibitively expensive, systematic flushing and bleeding was used to reduce levels below 40 μg/L, the Suggested Action Guide at the time. The maximum contaminant level for PCE is currently set at 5 μg/L.

PCE is a well-recognized animal and human neurotoxicant [3]. Many epidemiologic studies have reported mood changes, including increases in anxiety and depression among adults with occupational exposure to PCE and other solvents [4-10]. On the other hand, the published literature examining the effects of prenatal and childhood exposure to organic solvents on subsequent mental health is quite limited. To the best of our knowledge, early life exposure has been examined in only one prior study which found a 3.4-fold increased risk of schizophrenia (95% CI:1.3-9.2) among the offspring of parents who worked as dry cleaners [11]. We undertook a population-based retrospective cohort study to examine the long-term neurotoxic effects of prenatal and early childhood exposure to PCE contaminated drinking water. The current report focuses on the occurrence of four types of mental illness: depression, bipolar disorder, post-traumatic stress disorder, and schizophrenia.

## Methods

### Selection of Study Population

Individuals were eligible for the study if they were born from 1969 through 1983 to married women who lived in Cape Cod towns with VL/AC water pipes. Eight towns were involved --Barnstable, Bourne, Falmouth, Mashpee, Sandwich, Brewster, Chatham, and Provincetown--with one to 50 miles of affected pipes [2]. Eligible subjects were identified by cross-matching the maternal address from birth records with information from local water companies on the location and installation year of the pipes.

Based on a preliminary assessment, two groups of "index" subjects were selected: those initially designated as exposed at birth and those initially designated as unexposed at birth. Subjects who were initially designated as "exposed" had a birth residence that was either directly adjacent to a VL/AC pipe or adjacent to a pipe connected to a VL/AC pipe and the only possible water flow to the residence was through the VL/AC pipe (N = 1,910). Thus, the initial exposure status was based on a visual inspection of maps depicting the pipe distribution network in the immediate area of the birth address. A comparison group initially designated as "unexposed" was randomly selected from the remaining resident births during this period and frequency matched to "exposed" subjects on month and year of birth (N = 1,928).

In addition, 1,202 older siblings of the "index" subjects were identified for the present study. Only siblings born in Massachusetts during 1969-1983 were eligible for selection. All older siblings were initially considered "unexposed" at birth because they were born before the family moved to an affected Cape Cod residence. However, the initial exposure status of all subjects was considered provisional until more extensive exposure assessments, as described below, were completed.

Birth records were examined to gather information on each study family, including the name of the subject and his/her parents; the subject's birth date, birth weight and gestational duration; and the parents' ages and educational levels at the time of the subject's birth. All mothers were married when the "index" subject was born. The study was approved by the Institutional Review Boards (IRB) of the Massachusetts Department of Public Health and Boston University Medical Center and by the 24A/B/11B Review Committee at the Massachusetts Department of Public Health.

### Follow-Up and Enrollment of Study Subjects

Subjects were traced to obtain their current address and telephone number using Massachusetts Resident's Lists; death, and credit bureau records; telephone books, and the Internet White Pages. Letters were sent to all successfully traced subjects describing the general purpose of the study and requesting that they complete a self-administered questionnaire. Three follow-up letters were sent to non-respondents, and subjects who did not respond to these letters were phoned if their telephone number was available. As described in Table 1, 6.6% of the selected population could not be located (n = 332), 45.5% were located but never responded to numerous contact attempts (n = 2,294), 3.7% refused to participate (n = 187), and 2.2% were deceased (n = 111). In addition, the Massachusetts Department of Public Health IRB did not allow us to contact another 8.5% of subjects whose parent had refused to participate in our previous study of reproductive and developmental outcomes in this population (n = 427) [12]. These proportions were alike for both "exposed" and "unexposed" index subjects and their older siblings.

**Table 1:** Selection, enrollment, and initial and final exposure status of study subjects

	Initial Exposure Status			
	**Index Subject**		**Older Sibling**	

	**Exposed**	**Unexposed**	**Unexposed**	**Total**

Selected	1910	1928	1202	5040

Excluded during enrollment				

Deceased	35	40	36	111

Parent refused participation	199	148	80	427

Never located	113	149	70	332

No response	871	887	536	2294

Refused	73	78	36	187

Returned questionnaire	619	626	444	1689

Percent of selected	32.4%	32.5%	36.9%	33.5%

Percent of located	39.6%	39.3%	43.7%	40.5%

Excluded during exposure assessment				

Inadequate residential history	15	37	29	81

Off-Cape address in town with VL/AC pipes	19	27	50	96

Available for analysis	585	562	365	1512

Percent of selected	30.6%	29.1%	30.4%	30.0%

Final exposure status				

Both prenatal and early childhood exposure	561	160	110	831

Only early childhood exposure	7	42	85	134

Unexposed	17	360	170	547

Total	585	562	365	1512

Our comparison of available characteristics among participants and non-participants indicated that most characteristics were similar, including initial PCE exposure status (36.7% of participants vs. 38.8% of non-participants were exposed), age (mean age was 29.3 years for participants and 28.9 years for non-participants), race (98.5% of participants vs. 97.3% of non-participants were White), mean duration of gestation (40.1 weeks for participants vs. 39.9 weeks for non-participants), mean birth weight (3,428 grams for participants vs. 3,401 for non-participants), and birth order (47.7% of participants vs. 45.1% of non-participants were first born). Participants were more likely than non-participants to be female (60.7% of participants vs. 43.5% of non-participants), and have college-educated mothers (61.0% for participants vs. 49.3% for non-participants), but these differences were observed for both exposed and unexposed non-participants.

A self-administered questionnaire was sent to all successfully traced subjects to gather information on the subject's demographic characteristics including race, ethnicity, current marital status, educational level and occupation; personal history of chronic illnesses and obstetrical and gynecological problems; family history of mental disorders; occupational and non-occupational sources of solvent exposure; and residences from birth through 1990, including the exact street address and calendar years of residence for all Cape Cod addresses. Information was also collected on the occurrence four mental illnesses: depression, schizophrenia, bipolar disorder or manic-depressive disorder, or post-traumatic stress disorder (PTSD). In particular, subjects were asked if a doctor or health care provider *ever *said that they had the illness, and if the subjects responded affirmatively, they were asked for the year of diagnosis. Lastly, the survey gathered information on the subject's knowledge of the PCE contamination and self-assessments of their PCE exposure.

Additional data were also available for 81% of subjects whose mothers participated in our prior study on the impact of PCE exposure on reproduction and development. These data included maternal characteristics, such as changes in marital status; cigarette smoking, alcoholic beverage consumption, marijuana use, medical and obstetrical complications, and prenatal care during the subject's gestation; breast feeding practices; and exposure to solvents. We attempted to collect information on the mother's water consumption and bathing habits during the subject's gestation and the subject's consumption and bathing habits during childhood, but this information could not be recalled by a sizable portion of women and the prevalence of key variables was relatively low. Thus, these data were not incorporated into our present analyses.

### Geocoding of Residential Addresses

All reported residential addresses in the Cape Cod region were geocoded to a latitude and longitude using ArcGIS 8.1 by a team member (MER) who was masked to the exposure and outcome status of the subject. When possible, each address was geocoded to a specific parcel. Addresses that could not be geocoded to a specific parcel were geocoded to the closest parcel address by street number. If the street number was not available, the address was geocoded to the middle of the street when the street was less than one mile long or to the nearest intersection when the street was one mile or longer. Approximately 95% of reported addresses were geocoded. The remainder could not be geocoded because the information provided by the subject was insufficient.

### PCE Exposure Assessment

As described above, we assigned a tentative exposure status to subjects by visually inspecting maps of the pipe network in the immediately vicinity of the birth residence. To determine their final exposure status, we used a leaching and transport model of the entire distribution system to estimate the mass of PCE delivered to each residence from the prenatal period through the age of five years. The model, which was developed for our prior epidemiological studies by Webler and Brown [13,14], estimates the quantity of PCE entering the drinking water using information on the initial quantity of PCE in the liner (based on the pipe length and diameter), the age of the pipe, and the leaching rate of PCE from the liner into the water, as estimated by Demond [Demond AH: A source of tetrachloroethylene in the drinking water of New England: an evaluation of toxicity of tetrachloroethylene and the prediction of its leaching rates from vinyl-lined asbestos-cement pipe. MS Thesis. Massachusetts Institute of Technology; 1982].

The transport algorithm requires an estimate of water flow rate and direction, which depend on the configuration of the pipes and number of water users. The present study incorporated the Webler and Brown algorithm into the open source code of EPANET, water distribution modeling software which characterizes water flow throughout a distribution system. EPANET was originally developed by the US EPA to help water utilities conduct water quality monitoring [15], but has also been applied in several epidemiological studies assessing the health effects of drinking water pollutants [10,16-18].

Local water departments and the Massachusetts Department of Environmental Protection (DEP) provided information on the locations, installation dates and diameters of all VL/AC pipes in the public supplies. This information was used to create a schematic depicting the water sources, pipe characteristics, and nodes, representing points of water consumption along the pipe, and to assign each residence to the closest node on the distribution system. Because the study area was mainly comprised of residences, we assumed that all users drew the same amount of water. Available water company records also provided the basis for our assumption that water sources did not change during the study period.

The model integrated these data to simulate the flow of water through each network and to estimate the mass of PCE delivered to each node and all subject residences associated with the node for each year of the study period. We could estimate only annual PCE exposures because only move-in and pipe installation years were available. Thus, it was not possible to assess monthly variation in exposure. We estimated PCE exposure during the prenatal period by multiplying the annual mass of PCE that entered the subject's residence during their birth year by 9/12. We estimated cumulative exposure during early childhood by summing the mass of PCE that entered their residences from the month and year following birth through the month and year of the fifth birthday. Simple percentages were used to account for partial years.

We estimated PCE exposure levels only for subjects with complete geocoded residential histories from birth through age five. As shown in Table 1, 81 subjects were excluded because they had incomplete residential histories. Another 96 subjects were excluded because they had a residence in an off-Cape town with some VL/AC pipe and our PCE exposure assessments were limited to the Cape Cod region. We assumed that subjects who reported living in a Cape Cod town without any VL/AC pipes (n = 7) had no PCE exposure at that address because available records indicated little or no PCE contamination in areas without VL/AC pipes.

### Statistical Analysis

We compared the occurrence of each mental illness among subjects with prenatal and early childhood exposure combined to unexposed subjects. Because some subjects reported a history more than one mental illness, we examine each diagnosis among subjects with and without any other diagnoses. Nearly all subjects with prenatal exposure also had childhood exposure and so we were unable to examine the impact of prenatal exposure alone. In addition, we did not examine the impact of exposure only during childhood because there were too few subjects in this category (n = 134) to provide stable effect estimates.

The risk ratio (RR) was used to estimate the strength of the association between PCE exposure and the occurrence of each illness. Ninety-five percent confidence intervals were used to assess the precision of the risk ratios. Crude analyses were conducted and then generalized estimating equation (GEE) analyses were performed to account for non-independent outcomes arising from several children from the same family [19,20]. Thirty-nine percent of subjects were siblings. The logit link was used while assuming equal correlation between siblings.

Adjusted GEE analyses were conducted to assess the influence of confounding. Covariates considered for these analyses were demographic, medical and family characteristics, and non-drinking water sources of solvent exposure. These variables included the subject's gender, race, age, birth weight, gestational duration, history of chronic illnesses and obstetric/gynecologic problems predating the occurrence of any mental illness, and history of solvent-related jobs and hobbies; family history of mental illness (including depression, schizophrenia, bipolar disease and post-traumatic stress disorder among first degree relatives); the mother's age and educational level when the subject was born; paternal age, educational level and occupation when the subject was born; the mother's prenatal care, multivitamin use, alcoholic beverage consumption, cigarette smoking, marijuana use, medical conditions, and obstetrical complications when she was pregnant with the subject; breastfeeding history; number of live-born siblings and sibling deaths; and maternal history of solvent exposure. Each of these variables was added to the GEE model one at a time to assess the presence of confounding. However, none changed the crude estimates by more than 10% and so unadjusted GEE results are presented. Adjusted analyses were not attempted for schizophrenia because of the small number of cases (N = 4).

## Results

The study was based on 1,512 individuals who, according to the initial exposure assessment, included 585 exposed and 562 unexposed index subjects and 365 unexposed older siblings (Table 1). Approximately 27.4% of subjects (N = 414) changed their status during the detailed exposure assessment either because (1) their birth address was further downstream from a VL/AC pipe than was considered exposed in the preliminary visual assessment, (2) their modeled assessment indicated a different water flow direction than was originally assumed, (3) their questionnaire data indicated that they used a private wells, or (4) their questionnaire data indicated that they lived in an affected residence during childhood. Thus, the final analytic groups were comprised of 831 subjects with prenatal and early childhood exposure and 547 subjects with no exposure during either period.

As shown in Table 2, subjects were predominantly female, white, in their late 20s, college-educated, employed, and either married or cohabitating when they completed the study questionnaire. In addition, exposed and unexposed subjects had comparable proportions of personal characteristics such as occupational and hobby-related exposure to solvents, maternal characteristics such as smoking cigarettes and drinking alcoholic beverages during the subject's gestation, and familial characteristics such as a history of mental illness.

**Table 2:** Distribution of Selected Characteristics of Subjects and Parents by PCE Exposure Status

Characteristic	Both Prenatal and Early Childhood Exposure (N = 831)		Unexposed (N = 547)	
	**n**	**%**	**n**	**%**

Year of birth				

1969-1974	166	20.0	131	23.9

1975-1980	435	52.3	288	52.7

1981-1983	230	27.7	128	23.4

Current age (n, mean, sd)	831	29.2 (3.6)	547	29.6 (3.8)

Gender				

Male	331	39.8	216	39.5

Female	500	60.2	331	60.5

% White race	818	98.4	539	98.5

Current Educational Level				

High school graduate or less	128	15.4	67	12.2

Some college	192	23.1	144	26.3

Four year college grad or higher	510	61.4	335	61.2

Missing	1	0.1	1	0.2

Currently Employed				

Yes	719	86.5	487	89.0

No	92	11.1	54	9.9

Missing	20	2.4	6	1.1

Current marital status				

Single	272	32.7	157	28.7

Married or cohabitating	536	64.5	371	67.8

Other	19	2.3	12	2.2

Missing	4	0.5	7	1.3

History of chronic illness				

Yes	111	13.4	73	13.3

No	644	77.5	430	78.6

Missing	76	9.1	44	8.0

Ever had job with solvent exposure				

Yes	123	14.8	71	13.0

No	687	82.7	461	84.3

Missing	21	2.5	15	2.7

Ever had hobby with solvent exposure				

Yes	700	84.2	462	84.5

No	124	14.9	79	14.4

Missing	7	0.8	6	1.1

Mother's age at subject's birth (n, mean (sd))	831	27.2 (4.7)	547	27.5 (4.4)

Father's age at subject's birth (n, mean (sd))	831	29.8 (5.7)	547	29.8 (5.3)

Mother's educational level at subject's birth				

High school graduate or less	327	39.4	178	32.5

Some college	243	29.2	188	34.4

Four year college grad or higher	260	31.3	180	32.9

Missing	1	0.1	1	0.2

Father's occupation at subject's birth				

White collar	420	50.5	257	47.0

Blue collar	275	33.1	170	31.1

Other	126	15.2	112	20.5

Missing	10	1.2	8	1.5

Mother received prenatal care during subject's gestation				

Yes	794	95.5	520	95.1

No	4	0.5	0	0.0

Missing	33	4.0	27	4.9

Mother smoked cigarettes during subject's gestation				

11+ cigarettes a day	108	13.0	59	10.8

10 or fewer cigarettes a day	74	8.9	54	9.9

None	483	58.1	330	60.3

Missing	166	20.0	104	19.0

Mother consumed alcohol during subject's gestation				

1+ drinks a week	109	13.1	76	13.9

1-3 drinks a month	193	23.2	125	22.9

None	361	43.4	242	44.2

Missing	168	20.2	104	19.0

Mother smoked marijuana during subject's gestation				

Yes	25	3.0	18	3.3

No	640	77.0	420	76.8

Missing	166	20.0	109	19.9

Mother had medical and obstetrical complications during subject's gestation				

Yes	122	14.7	108	19.7

No	536	64.5	331	60.5

Missing	173	20.8	108	19.7

Mother had occupational exposure to solvents				

Yes	76	9.1	51	9.3

No	573	69.0	381	69.7

Missing	182	21.9	115	21.0

Subject's birth weight (n, mean, sd)	823	3,443 (506)	499	3,414 (534)

Subject's gestational age (n, mean, sd)	790	40.1 (2.5)	516	39.9 (2.4)

Multiple Pregnancy	21	2.5	20	3.7

Subject was breast fed				

Yes	406	48.9	299	54.7

No	254	30.6	140	25.6

Missing	171	20.6	108	19.7

Number of older siblings				

0	350	42.1	262	47.9

1	287	34.5	163	29.8

2+	193	23.2	119	21.8

Missing	1	0.1	3	0.5

Sibling died	16	1.9	15	2.7

Parent(s) divorced, separated, or died after subject's birth	51	6.1	32	5.9

Family history of schizophrenia				

Yes	10	1.2	4	0.7

No	800	96.3	536	98.0

Missing	21	2.5	7	1.3

Family history of post-traumatic stress disorder				

Yes	48	5.8	31	5.7

No	741	89.2	500	91.4

Missing	42	5.1	16	2.9

Family history of bipolar disorder				

Yes	66	7.9	39	7.1

No	727	87.5	497	90.9

Missing	38	4.6	11	2.0

Family history of depression				

Yes	323	38.9	199	36.4

No	458	55.1	321	58.7

Missing	50	6.0	27	4.9

Overall 22.2% of study subjects (336/1512) reported having one or more mental illnesses, and prevalence proportions were 17.5% (264/1512) for depression, 3.7% (56/1512) for bipolar disorder, 4.8% (73/1512) for PTSD, and 0.3% (4/1512) for schizophrenia. Four percent of subjects (n = 61) reported more than one mental illness; the most common combination of illnesses was PTDS and depression (n = 37). In addition, all four subjects with schizophrenia reported a second diagnosis: two reported depression and two reported bipolar disorder.

Analyses of the association between PCE exposure and reports of mental illness did not reveal any meaningful increases in the risk of depression among subjects exposed during gestation and early childhood (RR:1.1 for any exposure, 95% CI 0.9-1.4, Table 3). However, subjects with any exposure during gestation and childhood had a 1.8-fold increased risk of bipolar disorder (95% CI: 0.9-3.5), a 1.5-fold increased risk of post-traumatic stress disorder (95% CI: 0.9-2.5), and a 2.1-fold increased risk of schizophrenia (95% CI: 0.2-20.0). When exposure levels were examined, subjects in the highest exposure tertile had further increases in the risk ratios for bipolar disorder (RR 2.7, 95% CI: 1.3-5.6) and post-traumatic stress disorder (RR: 1.7, 95% CI: 0.9-3.2). While there were too few cases of schizophrenia to examine a dose-response relationship, it is notable that three of the four schizophrenia cases were exposed.

**Table 3:** Prenatal and Early Childhood Exposure to Tetrachloroethylene and the Risk of Mental Illness

Outcome	Exposure Category/Percentile	% Yes (n/N)	Crude Risk Ratio (95% CI)	GEE* Risk Ratio (95%CI)
				

Depression	Any	19.8 (152/769)	1.1 (0.9-1.4)	1.1 (0.9-1.4)

	> = 67^th^	19.0 (47/248)	1.1 (0.8-1.5)	1.1 (0.8-1.5)

	33^rd^-< 67^th^	20.8 (55/265)	1.2 (0.9-1.6)	1.2 (0.9-1.6)

	> 0-< 33^rd^	19.5 (50/256)	1.1 (0.8-1.5)	1.1 (0.8-1.5)

	None	17.8 (92/518)	Reference	Reference

				

Bipolar Disorder	Any	5.5 (36/653)	1.7 (0.9-3.2)	1.8 (0.9-3.5)

	> = 67^th^	8.2 (18/219)	2.6 (1.3-5.1)	2.7 (1.3-5.6)

	33^rd^-< 67^th^	3.2 (7/217)	1.0 (0.4-2.5)	1.1 (0.4-2.7)

	> 0-< 33^rd^	5.1 (11/217)	1.6 (0.7-3.5)	1.6 (0.7-3.7)

	None	3.2 (14/440)	Reference	Reference

				

Post-Traumatic	Any	7.1 (47/664)	1.5 (0.9-2.5)	1.5 (0.9-2.5)

Stress Disorder	> = 67^th^	8.2 (18/219)	1.7 (1.0-3.2)	1.7 (0.9-3.2)

	33^rd^-< 67^th^	6.7 (15/225)	1.4 (0.7-2.7)	1.4 (0.7-2.7)

	> 0-< 33^rd^	6.4 (14/220)	1.4 (0.7-2.6)	1.4 (0.7-2.6)

	None	4.7 (21/447)	Reference	Reference

				

Schizophrenia	Any	0.5 (3/620)	2.1 (0.2-20.0)	2.1 (0.2-20.0)

	> = 67^th^	0.5 (1/202)	--------	--------

	33^rd^-< 67^th^	0.9 (2/212)	--------	--------

	> 0-< 33^rd^	0.0 (0/206)	--------	--------

	None	0.2 (1/427)	Reference	Reference

*Generalized estimating equations

Similar results were observed when analyses of prenatal and/or early childhood exposure were limited to subjects who reported only one mental illness. For example, the risk ratios among subjects exposure during gestation and early childhood were 1.1 for depression (95% CI: 0.8-1.4), 1.5 for bipolar disorder (95% CI: 0.7-3.3), and 1.4 for post-traumatic stress disorder (95% CI: 0.5-4.0). (All four subjects with schizophrenia reported another diagnosis.)

However, no meaningful differences were observed for the age of diagnosis of each mental illness according to exposure status. In particular, the median ages at diagnosis among exposed and unexposed subjects were 21 and 22 years, respectively, for depression; 24.5 and 23 years, respectively, for bipolar disorder; and identical at 25 years for PTSD.

## Discussion

The results of this study suggest that the risk of certain mental illnesses is increased among individuals who were exposed to PCE-contaminated drinking water in early life. In particular, subjects with any exposure during gestation and early childhood had elevations in the risk of bipolar disorder and post-traumatic stress disorder that were further increased among subjects with the highest exposures. While the risk of schizophrenia was also elevated among exposed subjects, the number of cases was too small to draw reliable conclusions. In contrast, the risk of depression was not associated with prenatal and childhood PCE exposure.

The observed associations should be judged in light of the study limitations. First, the results are likely affected by exposure misclassification. Because historical exposure measurements were unavailable, we estimated the mass of PCE delivered to each subject's residence using EPANET water distribution modeling software that incorporated a leaching and transport model [13,15]. The model did not incorporate information on water consumption and bathing habits due to poor maternal recall of these behaviors. While the model also necessitated many assumptions about the water distribution system (for example, that all users drew the same amount of water), results from validation studies show good correlation between our exposure estimates and PCE concentrations in historical water samples (Spearman correlation coefficient (ρ) = 0.65, P < 0.00010) [21].

Because exposure misclassification was likely to be present for all subjects, regardless of their mental health status, the limitations in the exposure assessment likely attenuated the findings from dichotomous comparisons [22]. The direction of bias for comparisons involving the exposure tertiles is harder to predict, but risk ratios among subjects in the highest tertile are likely biased towards the null while those in the middle category may be biased either towards or away from the null.

Still another limitation arises the use of self-reports as the source of information on the mental illnesses. While subjects were asked if a health care provider ever said that they had each mental illness, it is unknown if the provider made the diagnosis based on criteria from the American Psychiatric Association's Diagnostic and Statistical Manual of Mental Disorders [23]. Furthermore, the prevalence of the illnesses among study subjects was slightly lower than that observed in general population surveys conducted in Massachusetts [24] and the United States [25,26]. For example, in 2001-2003 the lifetime prevalence of depression, bipolar disorder, and PTSD were 22.7%, 4.5% and 8.2%, respectively, in a nationally representative sample of adults aged 30-44 years [25].

Nonetheless, it is unlikely that inaccuracies in the diagnosis and reporting of the mental illnesses were related a subject's PCE exposure history because most subjects (and, by extension, their physicians) did not know this information. In particular, only 7% of subjects considered exposed by the modeled assessment believed that their drinking water was contaminated, whereas 29% believed that their water was not contaminated and 64% were unsure. Similarly, 31% of subjects considered unexposed by the modeled assessment believed that their drinking water was not contaminated while 5% believed that their drinking water was contaminated and 63% were unsure. The subjects' lack of knowledge about their exposure likely stems from their young age when the PCE contamination was publicized in the early 1980s.

A further limitation stems from possible residual confounding because of missing data on several risk factors for these mental illnesses, such as social supports and stressors [27]. However, in order to confound the associations observed in this study, these factors would need to be tightly correlated with PCE exposure, an unlikely circumstance given the uneven distribution of PCE contamination across Cape Cod. In fact, our current and prior studies of this cohort have found little or no confounding for the associations under investigation by variables that were collected [12,28].

Another limitation stems from the study's low response rate which reduced the statistical power of the study. It was also quite possible that individuals with mental illnesses, particularly severe mental illnesses, preferentially ignored our requests for participation and so reduced the number of cases in the final analytic population. However, available evidence suggests that this did not produce selection bias. First, similar proportions of participants and non-participants were exposed to PCE (36.7% of participants vs. 38.8% of non-participants). Second many characteristics of participants and non-participants were alike, including age, race, duration of gestation, birth weight, and birth order. Third, while participants were more likely to be female (60.7% of participants vs. 43.5% of non-participants) and have mothers with some college education (61.0% for participants vs. 49.3% for non-participants), these differences were present for exposed and unexposed non-participants. Lastly, losses stemming from the death of potential participants were small and unrelated to initial PCE exposure status (n = 111, Table 1). Also, our review of death records from the Massachusetts Registry of Vital Records and Statistics and the National Death Index found only one notation of a mental illness (acute psychosis induced by LSD) as a contributing cause of death.

PCE's potential to cause neurotoxic effects has been established through numerous animal and human studies [3]. Because PCE is a relatively small fat-soluble molecule, it easily crosses the blood brain barrier and has a high affinity for the lipophilic tissues of the central nervous system. While the mechanism by which PCE might cause neurotoxic effects is currently unknown [3], there is evidence to support mechanisms involving the peroxidation of cell membrane lipids [29], alterations in the fatty acid profile of the brain [30], and loss of myelin [31], and interactions with neuronal receptors [32].

High levels of prolonged occupational exposure to PCE and other solvents have been associated with chronic solvent induced encephalopathy [33] and, in some cases, schizophrenia among adults [34-36]. Solvent levels above the accepted threshold have also been associated with increases in anger and confusion among child workers aged 10-17 years [37]. Lower levels of occupational exposure have been also associated with increases in anxiety and depression among adults [4-8]. Because most of these occupational studies used employment information (such as job title and work practices) rather than industrial hygiene measurements to characterize individual exposure levels, it is difficult to compare directly the exposure levels in our population with those experienced by these worker groups. However, workplace settings typically result in higher exposure levels than environmental ones.

One community-based study of trichloroethylene (TCE)-contaminated drinking water also found higher levels of depression/dejection, confusion/bewilderment, and tension/anxiety among highly exposed adults; however, the effect was mainly attributable to exposed subjects who also consumed alcoholic beverages [10]. Highly exposed subjects in this study received drinking water with estimated TCE levels that were greater than 15 ppb (the uppermost value was not stated). In comparison, PCE levels in the drinking water supplies of our study population ranged from 1.5 to 7,750 ppb.

To the best of our knowledge, only one prior study has examined the impact of early life exposure to solvents on subsequent mental health. This study assessed the risk of schizophrenia among Israeli children whose parents worked in dry cleaning occupations by linking information on parental occupations from birth records with information on mental illness from a national psychiatric registry [11]. Four cases of schizophrenia were observed among 144 offspring with at least one parent working in dry cleaning (prevalence = 2.8%) for a crude relative risk of 3.4 (95% CI: 1.3-9.2). Adjustment for confounding factors did not appreciably alter these results.

In summary, the results of this study suggest that the risks of certain mental illnesses, particularly bipolar disorder, post-traumatic stress disorder, and schizophrenia are increased among adults exposed to PCE during gestation and early childhood. Independent investigations of similarly exposed populations are needed to corroborate these findings. Because PCE remains a commercially ubiquitous solvent and common contaminant of drinking water supplies [38,39], it is important to determine its impact on the health of vulnerable populations.

## Conclusions

While many studies of adults with acute and chronic solvent exposure have shown increased risks of anxiety and depressive disorders, there is little information on the impact of prenatal and early childhood exposure on the subsequent occurrence of mental illness. This retrospective cohort study examined whether early life exposure to tetrachloroethylene (PCE)-contaminated drinking water influenced the occurrence of depression, bipolar disorder, post-traumatic stress disorder, and schizophrenia among adults from Cape Cod, Massachusetts.

No meaningful increases in risk ratios for depression were observed among subjects with prenatal and early childhood exposure. However, subjects with any prenatal and early childhood exposure had elevated risks of bipolar disorder and post-traumatic stress disorder that increased further among subjects with the highest exposures. Thus, these results provide evidence against an impact of early life exposure to PCE on the risk of depression and evidence for an impact of early life exposure on the risk of bipolar disorder and post-traumatic stress disorder. The number of schizophrenia cases was too small to draw reliable conclusions. These findings should be confirmed in independent investigations of similarly exposed populations.

## Abbreviations

CI: Confidence Interval; DEP: Department of Environmental Protection; GEE: Generalized estimating equation; IRB: Institutional Review Board; PCE: Tetrachloroethylene; RR: Risk Ratio; VL/AC: Vinyl-lined asbestos-cement.

## Competing interests

Dr. David Ozonoff is Co-editor-in-Chief of Environmental Health. He has recused himself from all decisions involving the acceptance and publication of this manuscript. At the request of the Commonwealth of Massachusetts, in 1980 Dr. Ozonoff was a witness in bankruptcy court in a suit against the Johns-Manville Corporation, manufacturers of the ACVL water mains. He has also, on occasion, testified in personal injury and property damage cases involving exposure to tetrachloroethylene and trichloroethylene. Three years ago, Dr. Aschengrau served as a consultant in a personal injury case involving chlorinated solvent contamination. None of the parties in any litigation supported, reviewed or had knowledge of this paper. None of the other authors of this study have any competing interests.

## Authors' contributions

AA conceived the study and its design, coordinated data collection and analysis, and drafted the initial manuscript. JW, PAJ, TW, and VV provided technical input to data collection, exposure assessment, data analysis, and manuscript preparation. MER and LG conducted the data collection, geocoding, and exposure assessments. MRW and BRM participated in the data collection and conducted the data analyses. DO and RFW provided technical input to the study design, data collection, and manuscript preparation. All authors read and approved the final manuscript.
