# Correction to: Occurrence of mental illness following prenatal and early childhood exposure to tetrachloroethylene (PCE)-contaminated drinking water: a retrospective cohort study

**DOI:** 10.1186/s12940-020-00594-x

**Published:** 2020-04-09

**Authors:** Ann Aschengrau, Janice M. Weinberg, Patricia A. Janulewicz, Megan E. Romano, Lisa G. Gallagher, Michael R. Winter, Brett R. Martin, Veronica M. Vieira, Thomas F. Webster, Roberta F. White, David M. Ozonoff

**Affiliations:** 1 Department of Epidemiology, Boston University School of Public Health, Talbot 3E, 715 Albany Street, Boston, MA 02118 USA; 2 Department of Biostatistics, Boston University School of Public Health, Crosstown, 715 Albany Street, Boston, MA 02118 USA; 3 Department of Epidemiology, University of Washington, Box 357236, Seattle, WA 98195 USA; 4 Department of Environmental Health, Boston University School of Public Health, Talbot 4W, 715 Albany Street, Boston, MA 02118 USA; 5 Data Coordinating Center, Boston University School of Public Health, Crosstown, 715 Albany Street, Boston, MA 02118 USA; 6 Department of Neurology, Boston University School of Medicine, 72 East Concord Street, Boston, MA 02118 USA

**Correction to: Environ Health**

**https://doi.org/10.1186/1476-069X-11-2**

Following publication of the original article [1], the author reported that, because of a programming error, incorrect sentences and incorrect Table 3 has been published. The correct sentences and Table 3 are shown below.

1. In the Abstract, the following sentence is correct; “No further increases in risk were observed with increasing exposure levels.”
2. In the Results section, the following sentence is correct; “No further increases in risk were observed with increasing exposure levels.”
3. In the Conclusions section, the following sentence is correct; “However, subjects with any prenatal and early childhood exposure had elevated risk of bipolar disorder and post-traumatic stress disorder.”

**Table 3:** Prenatal and Early Childhood Exposure to Tetrachloroethylene and the Risk of Mental Illness

Outcome	Exposure Category/Percentile	% Yes (n/N)	Crude Risk Ratio (95% CI)	GEE^a^ Risk Ratio (95% CI)
Depression	Any	19.8 (152/769)	1.1 (0.9–1.4)	1.1 (0.9–1.4)
	> = 67th	19.5 (50/256)	1.1 (0.8–1.5)	1.1 (0.8–1.5)
	33rd- <67th	20.8 (55/265)	1.2 (0.9–1.6)	1.2 (0.9–1.6)
	> 0- <33rd	19.0 (47/248)	1.1 (0.8–1.5)	1.1 (0.8–1.5)
	None	17.8 (92/518)	Reference	Reference
Bipolar Disorder	Any	5.5 (36/653)	1.7 (0.9–3.2)	1.8 (0.9–3.5)
	> = 67th	5.1 (11/217)	1.6 (0.7–3.5)	1.6 (0.7–3.7)
	33rd- <67th	3.2 (7/217)	1.0 (0.4–2.5)	1.1 (0.4–2.7)
	> 0- <33rd	8.2 (18/219)	2.6 (1.3–5.1)	2.7 (1.3–5.6)
	None	3.2 (14/440)	Reference	Reference
Post-Traumatic Stress Disorder	Any	7.1 (47/664)	1.5 (0.9–25)	1.5 (0.9–2.5)
	> = 67th	6.4 (14/220)	1.4 (0.7–2.6)	1.4 (0.7–2.6)
	33rd- <67th	6.7 (15/225)	1.4 (0.7–2.7)	1.4 (0.7–2.7)
	> 0- <33rd	8.2 (18/219)	1.7 (1.0–3.2)	1.7 (0.9–3.2)
	None	4.7 (21/447)	Reference	Reference
Schizophrenia	Any	0.5 (3/620)	2.1 (0.2–20.0)	2.1 (0.2–20.0)
	> = 67th	0.0 (0/206)		
	33rd- <67th	0.9 (2/212)		
	> 0- <33rd	0.5 (1/202)		
	None	0.2 (1/427)	Reference	Reference

^a^GEE Generalized estimating equations
